# Supplementary material for: Electrophysiological priming effects demonstrate independence and overlap of visual regularity representations in the extrastriate cortex
Source: PLoS One. 2021 Jul 9;16(7):e0254361. doi: 10.1371/journal.pone.0254361 (PMC8270198; doi:10.1371/journal.pone.0254361)
Supplement: Attachment — Submitted filename: Response to Reviewers V4.docx [file pone.0254361.s001.docx]

PONE-D-20-34598
Electrophysiological priming effects demonstrate independence and overlap of visual regularity representations in the extrastriate cortex.
PLOS ONE

Dear Dr. Makin,

Thank you for submitting your manuscript to PLOS ONE. After careful consideration, we feel that it has merit but does not fully meet PLOS ONE’s publication criteria as it currently stands. Therefore, we invite you to submit a revised version of the manuscript that addresses the points raised during the review process.

Both reviewers agree that the current manuscript has merit, but have raised some questions about the design, analysis, and interpretation. Please address each of these thoroughly either by editing the manuscript and approach or rebutting reviewer points in a response. Note that reviewers will need to be satisfied with the methodological details (e.g., statistical analyses) and their interpretation before we can accept the manuscript, however I expect that it will be possible to do this sufficiently and thus am recommending a major revision.

We are grateful for this opportunity to resubmit our manuscript to PLOS ONE. The reviewers raise some legitimate points about statistical analysis. We have now produced an all-new "Supplementary materials 3" section with 5 tables of complete ANOVA analysis, as well as justification for treatment of familywise error rate on a case-by-case basis. We feel the complete analysis would be overwhelming if this were all in the main manuscript. Supplementary materials 3 is available on Open Science Framework (https://osf.io/2yjus/). We have also reproduced it at the end of this response to reviewer for convenience.

Changes to the manuscript are highlighted in yellow. Various minor improvements have been made. We re did the analysis for Experiment 5 after identifying a minor issue with the way one of the participants had been treated. This makes no substantial difference to the manuscript or figures.

Review Comments to the Author

Reviewer #1: The current paper presents the changes in the sustained posterior negativity (SPN) in response to the repetition of reflection/regularity stimuli. The authors claim that "SPN priming transferred between vertical and horizontal axis orientations (Experiment 4, N=48) and between reflectional and rotational symmetry". Large amount of work has been put in the manuscript and a number of experiments have been performed. Unfortunately, I have my concerns regarding the performed analysis and the interpretation of the results.

We are grateful for this careful reading of the manuscript. As described below, we have compiled a new Supplementary material 3, with complete ANOVA analysis. This is available on Open Science Framework (https://osf.io/2yjus/). We have also reproduced it at the end of this response to reviewers.

- No mentioning of a baselining of the data. Please, clarify if it was done and what was the window.

The ERPs were baseline corrected to a 200 ms pre-stimulus window (that is, the 200 ms before the onset of the first pattern). We have added this to the method section.

One problem with the original manuscript was that the 1500 ms pre-stimulus blank period is also sometimes referred to as the ‘baseline’. We have now changed this potentially misleading convention throughout.

- The analysis of three separate windows (first, second and third presentation) especially in the figures, make it difficult to compare the EEG responses to these stimuli. A better definition of SPN and its highlighting on the on the figures would be helpful.

In light of this comment, we have included a new figure in the introduction showing a textbook SPN from previous work (see below). We believe this helps readers appreciate properties of a typical SPN, and the foundations of the current paper. We have also added labels to subsequent figures.

***“Figure 1. Example Sustained Posterior Negativity from Palumbo et al. (2015).*** *A) Grand average ERP waves from posterior electrodes. P1 and the SPN intervals are labelled. B) SPN as a difference wave (reflection – random). Larger SPNs are ones where this difference wave falls a long way below zero. Here SPN amplitude scaled with the proportion of symmetry in the image. C) Example stimuli with 20 to 100% symmetry and corresponding topographic difference maps. Here the SPN appears as increasing blue at posterior electrodes. In the current series of experiments, we investigated how the SPN changes with repeated presentation.”*

- Part of the statistical analysis was done with rmANOVA but the degrees of freedom (DF) for it do not always match the number of observations or conducted comparisons. For instance, for mrANOVA, the following cannot be true: F(1.520,
71.437)=.... As far as I am aware, the DFs for rmANOVA are always an integer. Please, check it again and correct if necessary.

The reason for non-integer degrees of freedom is because the Greenhouse-Geisser correction factor was used to adjust them when the assumption of Sphericity was violated (Mauchly’s test, p < 0.05). We have now clarified this in the method section. This is a conservative move, which reduces false positive rates. On the downside, it leads to non-integer degrees of freedom. We have now clarified in the EEG analysis section.

- The significant interactions require further multiple comparisons with correction of p-values. It would help to better interpret the results.

It would be possible to follow up the significant interactions with many comparisons. Although complete statistical report is desirable in some cases, we feel that a ‘no stone unturned’ approach to statistical analysis would make the manuscript hard to read. We have thus included the complete ANOVA output in our new Supplementary material 3. This allows readers to see the full picture with the aid of 5 tables. This also discusses decisions about correction for multiple comparisons. The important SPN priming effects survive correction for multiple comparisons.

It is available on Open Science Framework (https://osf.io/2yjus/). We have also reproduced it at the end of this response to Reviewers.

- I don't think using only 64 channel EEG for source localization is enough for making sound claims about the location of the effect (especially the ones that can go into the title). I would be more careful with the interpretation of the source localization data.

We agree that source localization probably requires more than 64 channels. However, our claims about SPN priming happening ‘in the extrastriate symmetry network’ in the title are not based on this source localization analysis alone. We have now explained more in the Discussion:

*“We acknowledge that source localization is insufficient to support an original claim about neuroanatomy, especially when it based on just 64 electrodes. However, converging evidence from previous fMRI, TMS and EEG studies make us confident that the SPN is generated in the extrastriate cortex. We do not need the new source localization analysis to be confident that the SPN is generated in the extrastriate cortex. However, we do need source dipole analysis to establish that SPN priming recorded on the scalp actually happens within the extrastriate cortex. Without the source dipole analysis, our interpretation of SPN priming would be ambiguous.”*

- In the experiment 1 it is mentioned that the data from grand-average of posterior electrodes was chosen, why not use the entire scalp and separate it in ROIs. That way one can see better a "spatial" distribution and development of the effect.

We are not sure this would improve on the LIMO analysis already included in Supplementary materials 2. Indeed, it would add another factor to the already-complicated ANOVAs. We feel LIMO provides an efficient snapshot of spatial distribution. We discuss this in the Results of Experiment 1:

*“This basic ERP analysis was based on a subset of electrodes and time windows. Rather than running whole scalp analysis with Electrode cluster as an additional factor, we complemented the above with mass univariate and global field power (GFP) analysis, which incorporates all electrodes and time points. This is a more efficient way of imaging the full data set, and gives comparable results (see supplementary materials 1, https://osf.io/2yjus/).”*

- Was a speeded response or a delayed response used for the task?

Participants entered their responses in an unspeeded way after the end of the third presentation. We have now clarified this in the method.

- The authors claim that they used an implicit task to avoid the repetition effect, but this effect can be observed without an explicit task and especially in the later experiments, the spatters (the random images) were starting to resemble faces. Since our cognition is prone to recognizing faces everywhere, how would authors deal with the issue of changes in N170 (an ERP negativity responsive to face processing) that temporally should overlaps with SPN.
- In the same vein, did the authors asked their participants if they recognized some pictures in the random patterns? The behavioural response would exclude the possibility of subjects giving a semantic background to their stimuli and therefore would avoid other type of priming.

Unfortunately, we did not systematically interview the participants about their subjective associations. It is true that some patterns may have resembled faces and generated an N170. However, face responses would be much more pronounced in 1-fold vertical reflection conditions. If the SPN priming effect had been unique to conditions with 1-fold vertical reflection, it might be explained by increasing activation of face sensitive regions (such as the OFA and FFA). However, SPN priming was also found in many other conditions, such as Horizontal reflection, and Rotational symmetry, and 8-fold reflection, where face pareidolia is less likely. We have now added this to the General discussion:

*“Finally, abstract symmetrical patterns might be perceived as faces (a case of face pareidolia). However, it is unlikely that activation of face-sensitive visual areas with repeated presentation is responsible for SPN priming. The face response would be strongest for 1-fold vertical reflection in Experiment 4, and SPN priming was not unique to this condition.”*

- Why were the oddball results not included in the study? It would have been interesting to see if the priming effect stands after the interruption with the middle blank stimulus.

This is a good idea. However, the oddball trials always involved the sequence ‘random – blank – random’ so there was no SPN on oddball trials. We are quite confident that specifics of the oddball task were not responsible for our SPN priming effect. After all, SPN priming was found by Bertamini et al. (2019), where all aspects of the experiment were quite different. We have now added this in discussion of Experiment 1.

*“Another recent SPN study by Bertamini, Rampone, Oulton, Tatlidil, & Makin (2019) found comparable results. In this experiment two patterns were separated by a one second ISI. The SPN generated by the second pattern was larger in the category repeat condition (different examples of reflectional symmetry) than in the exemplar repeat condition (identical examples of reflectional symmetry). This again shows that SPN priming only happens when novel exemplars are repeated. The fact that this experiment involved different task and stimuli provides good converging evidence for selective SPN priming. ”*

- Especially that the behavioural results were quite high, why not exclude the EEG trials with wrong behavioural results. This way, the data would have been better controlled.

We can see why this would often be a good idea. However, we do not think this would make the data ‘better controlled’ in our case. Note that response mapping switched unpredictably between trials. On some trials participants pressed with their right hand to report ‘All patterns’ and with their left hand to report ‘Blank in middle’. On other trials response mapping was reversed. This hand-switching was moderately demanding. It often involved inhibiting habitual responses built up over a few trials. It is likely that most errors were introduced at the button selection stage, and do not reflect genuine perceptual confusion about whether there was a blank in the middle or not. Removing rare error trials thus means removing trials where visual processing was adequate. This would reduce signal to noise ratio (slightly). We have now explained this in the method.

*“Trials were included in EEG analysis even if participants gave the wrong behavioural response. Given the moderate cognitive demands of the unpredictable key mapping, it is likely that most errors were introduced at the response entry stage. Most errors are unlikely to reflect genuine confusion about whether there was a blank in the middle.”*

- In the discussion on experiment 1, the authors claim: "It seems that there is something special about the onset of new reflection exemplars, which increases activation of the extrastriate SPN generators." Have you thought of a surprise effect? Breaking of a regular pattern can lead to the change in EEG-response around 250 ms post-onset (mismatch negativity) which would also co-occur with SPN.

This is a possibility, but the surprise effect would also be present when three different random patterns are shown in sequence. However, the identical random and different random sequences produce indistinguishable waves. We have now added this to discussion of Experiment 1:

*“Second, the different exemplar sequences might evoke cognitive surprise. Indeed, several ERPs, such as the Mismatch Negativity, are generated when expectations build up over a sequence of events and are then violated (e.g. Naatanen, Paavilainen, Rinne, & Alho, 2007). However, such non-specific expectation effects are subtracted when the SPN is computed as a difference wave (different reflections – different randoms). It is also noteworthy that different random and identical random waves were very similar (Figure 3A), which is inconsistent with cognitive surprise explanations. ”*

- The source localization part on experiment 2 should go into supplementary materials since it does not provide any statistically relevant results.

We assume the reviewer is referring to the rear-view topographies here. There was no source localization in Experiment 2. We have now moved this to Supplementary materials.

- Please refer to all the results with p-value above 0.05 as statistically non-significant.

We agree. We have now removed terms like ‘marginally significant’ and ‘borderline significant’.

- Please specify the total number of ICAs out of which you mentioned how many you removed.

There were 64 ICA components. We have now added this.

- The authors claim: "we have previously found that large alterations to band pass filters, ICA or trial exclusion rules do not change the shape of SPN wave dramatically". I would do this claim since even though the shape of SPN might not change, its variability might change drastically due to considerably decreased SNR. This would very much influence your results on statistical analysis.

We agree that this is a possibility. We have removed this potentially misleading section from the EEG analysis.

- Please change the figures with ERP presentation so that the comparison between the SPNs are more clear and that the reader can better distinguish between the ERP components.

We have reconsidered and relabelled the figures (see new Figure 3 below with red label in A below). Furthermore, we have also added the new Figure 1 which hopefully helps the reader see SPNs in the other figures.

***“Figure 3. Experiment 1 results****.* ***A)*** *Grand Average ERPs from electrode cluster O1, PO7, O2 and PO8. Colour corresponds to coloured outlines in Figure 1. Results are averaged over the Complex and Simple patterns.* ***B)*** *The SPN shown as a difference wave. The intervals where the patterns were visible on the screen are shown with dotted boxes.* ***C)*** *Mean SPN for first, second and third intervals. Note SPN priming in the different reflections condition (red), but not in the identical reflections condition (dark blue). Results from Experiment 1. Error bars = 95% confidence intervals (if they do not cross zero, the amplitude difference from zero significant at the .05 level). Stars show significant pairwise comparisons within an interval (* p <0.05, *** p <0.001).”*

Please advise if you think more changes to Figures are needed.

**Reviewer #2:**

In this paper, Makin et al. measured the ERP for a sequence of symmetric or random patterns. They studied how the SPN changed with various stimulus parameters. The result can help to establish an ERP paradigm that discerns whether two stimulus parameters are processed by the same symmetry processing mechanisms. In general, this paper is well written and the result is useful. However, some issues need to be addressed before publication.

Statistics. The use of post-hoc statistics was not problematic. For instance, in Exp 1 (P. 8), the authors first performed a three-way ANOVA, followed by a two-way ANOVA, and then a one-way ANOVA on the third SPN and a t-test. There are several issues here. The first three-way ANOVA is obviously the omnibus ANOVA for the whole experiment and thus all the subsequent analyses should be considered as its post-hoc analyses. Since the authors found significant effects from sequence type, sequence position, and their interaction, the next step should be testing the sequence type effect at different sequence positions or vice versa. So, the one-way ANOVA and the t-test at the end are in the right direction, but the two-ANOVA is out-of-place. It provides no extra information from the three-way ANOVA and was misleading and thus should be removed. Even the one-way ANOVA and the t-test have their own issues. As post-hoc analyses, their error term should be the same as the omnibus ANOVA. Thus, why the degree-of-freedom for MSE of the ANOVAs and the t-test in these tests are different? Furthermore, the correction of significance level for multiple comparisons was not reported. Similar issues also exist for other experiments.

*we assume you mean ‘The use of post-hoc statistics **was** problematic’ here.

Our original Experiment 1 ANOVA report involved some behind-the-scenes decisions which were not transparently explained. To correct this weakness, we have provided a new Supplementary material 3 with complete ANOVA analysis of all experiments, and further exposition. We hope our new Supplementary materials 3 addresses your concerns. This is available on Open Science Framework (https://osf.io/2yjus/). We have also reproduced it at the end of this response to reviewer’s document.

We can see why reporting just one of three possible 2X3 sub-ANOVAs would seem problematic and incomplete. We were perhaps unclear about our specific motivations for focusing on *this particular* 2X3 ANOVA, rather than the other two. In this case, the difference between Different reflections and Identical Reflections is especially interesting. The other two possible 2X3 ANOVAs (comparing against the RandRandRef condition) are less informative. They are highly significant, but this is because of the unsurprising absence of an SPN in RandRandRef until Sequence Position 3, where we are comparing random with random. We have now clarified this in Supplementary materials 3 (https://osf.io/2yjus/).

One reason the DF in the omnibus ANOVA and t tests seem mismatched is because the ANOVA DF were adjusted due to violations of the assumption of sphericity (a conservative approach). The second reason is that the between-subjects factor Stimulus Type (Simple, Complex) is included in the mixed ANOVAs, but not in follow up tests. We have now clarified this in the method and results.

P. 11 First paragraph. Here, the authors rejected two alternative explanations of their data. However, these should be elaborated. For instance, it is not obvious to me why “If the priming effect was inherited from lower visual areas (Feuerriegel, 2016), it would have been [sic] stronger in the identical reflections condition”. The weaker response for the identical reflections condition might come from adaptation and has nothing to do with whether the effect is from the lower visual areas or not.

Our reasoning here was admittedly speculative. Given that the paper is already complicated, we have removed this rather than elaborating further.

P. 13. Exp. 2 Result. It is odd that the authors chose to average the left and right hemisphere electrodes while the stimuli were presented in either left or right visual fields. Why not set two mosaics of electrodes, one for the left and the other for the right hemispheres, and analyze them separately. In this way, the authors should be able to detect strong priming effects.

There are subtle reasons why combining both hemispheres were the most straightforward approach to analysis of Experiment 2. We did not explain this sufficiently in the paper.

There are two ways SPN priming could manifest in Experiment 2. It could be that there is an SPN builds up over the contralateral hemisphere (opposite the symmetry) exclusively.

However, it could be that SPN priming partly happens over the ipsilateral hemisphere. For example, priming may involve more sharing of symmetry information between hemispheres with repeated presentations. This may happen more in the changing locations condition. We would not want to bias against unexpected SPN priming in the changing locations condition by picking and analysis that ruled out this alternative.

We also saw advantages to using the same 3X2 design with the same electrodes in every experiment (there was a mistake with Figure 6 legend suggesting Experiment 2 was analysed with alternative electrodes -we have now corrected this).

However, we have included substantial extra discussion and analysis separated by hemisphere in the Supplementary materials 3 (see reproduction of Supplementary materials 3). Although this analysis by hemisphere was not very conclusive, it demands another paragraph in Discussion of Experiment 2.

*“Nevertheless, these results are complicated slightly by an ipsilateral contribution to SPN priming in the repeated locations condition (as described in Supplementary materials 3). Specifically, when reflection was repeated the left hemifield, there was SPN priming in the contralateral right hemisphere (as expected), but also increasing negativity in the ipsilateral left hemisphere (not expected). This could be explained if participants broke central fixation and moved their eyes to the symmetry on the left side of the screen. However, eye tracking provided no evidence for this (Supplementary materials 2). It would be a mistake to overinterpret this unexpected aspect of the results. ”*

Experiment 3 and 4. By the texts on P. 15 and 17, it seems that Exp 3 and 4 were almost identical. However, the stimuli presented in Fig. 7 and 9 were different, one set contained oblique patterns while the other vertical and horizontal. Which one is right? Also, even the authors did use patterns with different orientations in Exp 3 and 4, they still have to make clear the motivation of such two very similar experiments and to contrast the differences between them.

We apologize that the distinction between Experiment 3 and 4 was not sufficiently clear in the original manuscript. Experiment 3 had orientations anywhere between 0 and 90 deg (so often oblique), and Experiment 4 had just horizontal and vertical reflections. We have now added to the discussion of Experiment 3 to make the results more distinct.

*“Despite the apparent orientation-selectivity of SPN priming in Experiment 3, we cannot claim SPN priming is completely orientation selective without examining transfer between horizontal and vertical reflections. After all, horizontal and vertical axes have a special visual status (e.g. Appelle, 1972) and horizontal and vertical reflections are more salient in many conditions (Wenderoth, 1994). This was the topic of Experiment 4. ”*

.

**BELOW IS THE NEW SUPPLMENTARY MATERIALS 3.**

*******************************************************************************

**Supplementary Materials 3. Complete ANOVA analysis**

Some analysis decisions were not explained and justified in the manuscript for the sake of brevity. This supplementary materials section provides the necessary elaboration and full analysis.

**Experiment 1**

As explained in the main manuscript, Experiment 1 had a 3X3X2 mixed design. There were two within-participant factors [3 Sequence position (first, second, third) X 3 Sequence type (different reflections, identical reflections, RandRandRef)] and one between-participant factor [Complexity (simple, complex)]. Analysis from Experiment 1 is shown in Table 1. Figure 1 shows results again to facilitate comparison (collapsing over the between participant factor Complexity).

The 2X3 ANOVA comparing different and identical reflection sequences is of particular theoretical interest. The significant effects here survived correction for multiple comparisons (i.e. p X number of tests in the ANOVA < 0.05). There are two other 2X3 ANOVAs from Experiment 1, comparing identical reflections with RandRandRef and comparing different reflections with RandRandRef. These also reveal significant Sequence position X Sequence type interactions, but this is due to the unsurprising absence of an SPN in RandRandRef until sequence position 3. They are included in Table 1 for completeness.

The pairwise comparison between Third SPN in different and identical reflections conditions was theoretically interesting, because it establishes selectivity of SPN priming. This was one of three pairwise comparisons. One could multiply p = 0.011 by 3 to give 0.033. However, a more conservative approach would be to multiply p by 9 (given that there are 9 tests including the those from the original 2X3 ANOVA). This gives 0.099, which is not significant with a two-tailed test.

***Table 1. Complete analysis of SPNs in Experiment 1.*** *Effects that would survive correction for multiple comparison are shown in red. Other significant effects are pink. As in the manuscript, non-integer degrees of freedom result from application of the Greenhouse-Geisser correction factor.*

***Figure 1. Results from Experiment 1.*** *Error bars = 95% confidence intervals (if they do not cross zero, the amplitude difference from the randoms sequence is significant at the .05 level). Stars show significant pairwise comparisons within an interval (* p <0.05, ** p < 0.01, *** p <0.001).*

**Experiment 2**

Table 2 and Figure 2 show results from Experiment 2. None of the significant effects in would survive correction for multiple comparison here. However, we predicted a selective SPN priming effect in the repeated locations condition of Experiment 2. It is thus justifiable to use uncorrected p values when the Sequence position X Sequence type interaction, and the effect of Sequence position in the repeated locations condition.

There was also an unexpected effect of Sequence position in the changing locations condition. However, this was not a case SPN priming, but unexpected positivity at sequence position 2 (a quadratic rather than linear effect).

Our interpretations of Experiment 2 would be more completely confirmed if there were significant difference between repeated and changing locations at sequence position 3. This was not significant with at two tailed test (p = 0.069). Instead, the only significant pairwise comparison was at sequence position 2, and this was partly driven by the unexpected positivity in the changing locations sequence.

***Table 2. Complete analysis of SPNs in Experiment 2.*** *Conventions are the same as Table 1.*

***Figure 2.*** *Results from Experiment 2. Conventions are the same as Figure 1.*

**Experiment 2 breakdown by hemisphere**

In Experiment 2, SPN priming may not necessarily be restricted to the contralateral hemisphere. It is conceivable that repeated presentation of symmetry on one side could also involve increased negativity over both ipsilateral and contralateral hemispheres. Here we include more detailed breakdown by hemisphere.

Results from the consistent locations condition are shown in Figure 3, with ERPs from left (P3 P7 PO3 and PO7) and right (P4, P6, PO4 and PO8) hemispheres separately. These electrode clusters were selected post hoc to best capture the left and right hemisphere SPNs (unlike the original analysis, that used a priori electrode cluster consistent with the other Experiments). Figure 3 reveals that priming was not limited to the contralateral hemisphere – amplitude at the ipsilateral hemisphere also became slightly more negative from presentations 1 to 3 as well (particularly over the left hemisphere when reflection was on the right, Figure 3C). This suggests that the SPN spread between hemispheres when patterns were presented at a consistent location. This unexpected effect requires further research, but we stress that it is NOT akin to SPN priming between changes in retinal location. Mixed ANOVA confirmed the Hemisphere X Hemifield interaction (F (1,46) = 19.077, *p* < .001, η_p_^2^ = .293). However, the main effect of Sequence Position (F (1.696, 78.003) = 4.079, *p* = .026, η_p_^2^ = .081) did not interact with Hemisphere (F (1.512, 69.530) = 0.643, *p* = .487). There were no other effects or interactions (*p* > .136)

Next, we focus on the results from the changing hemispheres condition. The reflections still generated contralateral SPN, but this alternated from left to right. One interesting but unexpected result was obtained from the second presentation: Here Random generates a contralateral positivity compared to the double random condition. These results are shown in ERP waves in Figure 4. The alternating contralateral SPN was confirmed by a significant Hemisphere X Sequence Position X Sequence Type interaction (F (1.641, 75.941) = 66.038, *p* < .001, η_p_^2^ = .589). Unlike all other effects reported above, this was modulated by the between factor Complexity (F (1.641, 75.941) = 5.344, *p* = .010, η_p_^2^ = .104). The three-way interaction was stronger when patterns involved simple, larger elements (F (1.548,35.595) = 58.968, *p* < .001, η_p_^2^ = .719) than a complex combination of large and smaller dots (F (2,46) = 15.778, *p* < .001, η_p_^2^ = .407). This was perhaps because the simple patterns were more salient in the visual periphery.

In summary, this exploratory breakdown by hemisphere confirms that the SPN priming effect is unique to the Repeated locations condition (Figure 3) and not found in the changing locations conditions (Figure 4). However, one caveat is that the SPN priming in repeated locations condition is partially driven by increased ipsilateral negativity in the RefRand condition (maroon in Figure 3A-C). This can also be seen in the rear-view topoplots in Figure 4 of Supplementary materials one. One obvious explanation for this is that participants disobeyed task instructions and fixated on the left side of the screen when a reflection pattern was repeated in that location. This would produce a bilateral SPN. However, this problematic alternative explanation is not consistent with Eye tracking analysis (Supplementary materials 2), which would have identified systematic fixation breaks.

***Figure 3. Experiment 5 results from each hemisphere (repeated locations conditions)****. ERPs from the left posterior electrodes (P3, P5, PO3 PO7) are shown on the left (A-C), and right posterior electrodes (P4, P4, PO4, PO8) on the right (D-F). RefRands = Reflection patterns on the left of fixation, and random on the right of fixation. RandRefs = Random on the left, and reflection on the right. The contralateral SPN is most obvious in the bar charts in C and F. Conventions are the same as Figure 1.*

***Figure 4. Experiment 2 results from each hemisphere (changing locations conditions)****. REFlREFrREFl refers to conditions where reflection shifted left to right to left. REFrREFlREFr refers to conditions where reflection shifted right to left to right. Conventions are the same as Figure 1.*

**Experiment 3.**

Table 3 and corresponding figure below show the complete results of Experiment 3. As stated

in the manuscript, the expected significant effect of Sequence position in the repeated orientations conditions must be treated with caution due to the lack of interaction.

**Table 3. Complete analysis of SPNs in Experiment 3**. Conventions are the same as Table 1.

***Figure 5.*** *Results from Experiment 3. Conventions are the same as Figure 1.*

**Experiment 4**

Experiment 4 has no statistical ambiguity. There was a main effect of Sequence position only, and this would survive correction for multiple comparisons.

***Table 4. Complete analysis of SPNs in Experiment 4****.* Conventions are the same as Table 1.

***Figure 6. Results from Experiment 4.*** *Conventions are the same as Figure 1.*

**Experiment 5**

Although there are some caveats regarding mass univariate and source dipole analysis for Experiment 5 the standard SPN analysis was statistically unambiguous. There was a main effect of sequence position only, and this would survive correction for multiple comparisons.

***Table 5. Complete analysis of SPNs in Experiment 5****.* Conventions are the same as Table 1.

***Figure 7. Results from Experiment 5.*** *Conventions are the same as Figure 1.*
